# Vascular Tissue Engineering: Polymers and Methodologies for Small Caliber Vascular Grafts

**DOI:** 10.3389/fcvm.2020.592361

**Published:** 2021-01-11

**Authors:** Bruna B. J. Leal, Naohiro Wakabayashi, Kyohei Oyama, Hiroyuki Kamiya, Daikelly I. Braghirolli, Patricia Pranke

**Affiliations:** ^1^Hematology and Stem Cell Laboratory, Faculty of Pharmacy, Universidade Federal Do Rio Grande Do Sul, Porto Alegre, Brazil; ^2^Post-graduate Program in Physiology, Universidade Federal Do Rio Grande Do Sul, Porto Alegre, Brazil; ^3^Division of Cardiac Surgery, Department of Medicine, Asahikawa Medical University, Asahikawa, Japan; ^4^Stem Cell Research Institute, Porto Alegre, Brazil

**Keywords:** vascular tissue engineering, cardiovascular disease, vascular graft, electrospinning, polymers

## Abstract

Cardiovascular disease is the most common cause of death in the world. In severe cases, replacement or revascularization using vascular grafts are the treatment options. While several synthetic vascular grafts are clinically used with common approval for medium to large-caliber vessels, autologous vascular grafts are the only options clinically approved for small-caliber revascularizations. Autologous grafts have, however, some limitations in quantity and quality, and cause an invasiveness to patients when harvested. Therefore, the development of small-caliber synthetic vascular grafts (<5 mm) has been urged. Since small-caliber synthetic grafts made from the same materials as middle and large-caliber grafts have poor patency rates due to thrombus formation and intimal hyperplasia within the graft, newly innovative methodologies with vascular tissue engineering such as electrospinning, decellularization, lyophilization, and 3D printing, and novel polymers have been developed. This review article represents topics on the methodologies used in the development of scaffold-based vascular grafts and the polymers used *in vitro* and *in vivo*.

## Introduction

Cardiovascular disease (CVD) is a group of diseases related to the heart and vasculature including coronary, cerebral, and peripheral artery disease (1, 2), and was responsible for 15.2 million deaths in 2016 according to the World Health Organization, being the major cause of death throughout the world (3). Atherosclerosis is the most common predisposition of CVD and the damage to blood vessels can provoke morphological changes such as stenosis, occlusion, or dilation, causing malperfusion of end-organs or rupture of vascular walls (4, 5). Despite pharmacotherapy using vasodilators or anticoagulants being the first-line treatment for symptomatic patients, surgical repair is an indispensable treatment option in patients with advanced diseases, and replacement or bypass of diseased vessels with vascular grafts is generally performed regardless of the pathological or anatomical categories above (6).

### Middle and Large-Caliber Grafts

Because of the absence of alternative autologous grafts and an adequate patency rate, large-caliber (10–30 mm) synthetic grafts have predominantly been developed. Bakey reported the first successful synthetic vascular graft implantation in human using a polyester (polyethylene terephthalate: PET, named Dacron) vascular graft in 1954 (7). Since then, through a large number of trials with a variety of materials, PET (Dacron®), expanded polytetrafluoroethylene (ePTFE), and polyurethane (PU) are clinically approved nowadays (Table 1) (14). These materials are mechanically and biologically compatible with native blood vessels. In addition to constant material improvement, accessorial modifications have been developed such as heparin conjugation on a luminal surface to append anticoagulative function or ring attachment to prevent the collapse of a graft; these improvements are also contributing to a long-term patency in middle (5–10 mm)-caliber grafts (15). As a result, the synthetic vascular grafts demonstrate satisfactory outcomes in the repair of large and middle-caliber vasculatures.

**Table 1:** Vascular grafts approved for clinical use: summary of the vascular grafts currently approved and commercially available for clinical use based on U.S. and European guidelines of revascularization (8–11).

**Vascular size**	**Clinical disease**	**Surgical repair**	**Autologous graft**	**Synthetic graft**		
				Material*	**Diameter**	**Accessory processing**
Large	Aortic aneurysm Aortic dissection	Replacement	Not available	Polyethylene terephthalate (Dacron) (7) Expanded polytetrafluoroethylene (ePTEE) (12) Polyurethane (13)	18–30 mm	Bovine gelatin sealing of outer surface (leakage prevention) Heparin coating of inner surface (anticoagulation) Layering (elastic and hemostatic effect) Ring support (kink and collapse prevention)
Middle	Carotid artery disease Lower-extremity Arterial disease (above knee) Arteriovenous fistula	Replacement Bypass Patch plasty	Great saphenous vein Upper-extremity vein		5–10 mm	
Small	Coronary artery disease Lower-extremity Arterial disease (below knee)	Bypass	Intra thoracic artery Great saphenous vein Radial artery Gastroepiploic Artery	Not available		

Variety of synthetic grafts are available for mid to large vascular repair and the smallest diameter of a commercialized graft for revascularization is 5 mm. In contrast, autologous grafts are the only option for small caliber vascular repair. *References indicate the first reports in human use.

### Small-Caliber Grafts

Within CVD, ischemic heart disease (IHD) has been the leading cause of death for decades; over 9.4 million people died in 2016 throughout the world (3). In addition, an increasing number of patients have peripheral artery disease (PAD) due to the aging of the society and changing lifestyles (16). In advanced cases of these pathologies, the patients require surgical revascularization such as coronary artery bypass grafting (CABG) or peripheral artery bypass grafting. The grafts clinically used for these small-caliber (<5 mm) revascularization rely exclusively on autologous grafts because no small-caliber synthetic grafts are currently approved for clinical use due to their much lower patency rate than autologous grafts (Table 1) (17). There are, however, several limitations to autologous grafts, such as harvesting invasiveness, poor quality (excessively thin or thick), or quantitatively inadequate availability in patients with severe systemic atherosclerosis or in those whose vessels have already been harvested for previous surgeries (18, 19). Even though the autologous vessels represent the current gold standard for vascular grafts, complications causing graft failure can occur and additional grafts for surgical reintervention are needed (18, 20). Thus, an alternative, practical to use, small-caliber vascular graft is in high demand.

The major factors of failure in non-autologous small-caliber grafts are acute thrombogenicity, intimal hyperplasia and infection. Endothelial cells (ECs) play an important role in antithrombogenicity by suppressing the activation of platelets and following coagulation cascades in native vessels (19); therefore, the lack of ECs in a graft causes thrombosis, leading to stenosis and occlusion of the vascular lumen (21). Intimal hyperplasia is a significant factor of long-term patency. Although the exact mechanism is still unclear, excessive proliferation of smooth muscle cells is deemed to be a cause. In addition, compliance (elasticity) mismatch or undistributed wall stress may cause fibrous proliferation and intimal hyperplasia around the anastomosis line (22). A permanently implanted foreign body is a common cause of devastating infections, which can be a factor of failure in non-autologous grafts (23). In order to overcome these problems and create a clinically practicable small-caliber graft, innovative methodologies have been developed for vascular tissue engineering such as electrospinning, decellularization, lyophilization, and 3D printing.

The present review has therefore aimed to describe the main techniques and polymers which have been used for the production of scaffold-based vascular grafts by tissue engineering (TE). In addition, the main results that had already been obtained in *in vivo* studies of vascular tissue engineering (VTE) have been highlighted.

## Vascular Tissue Engineering

**Figure 1:**
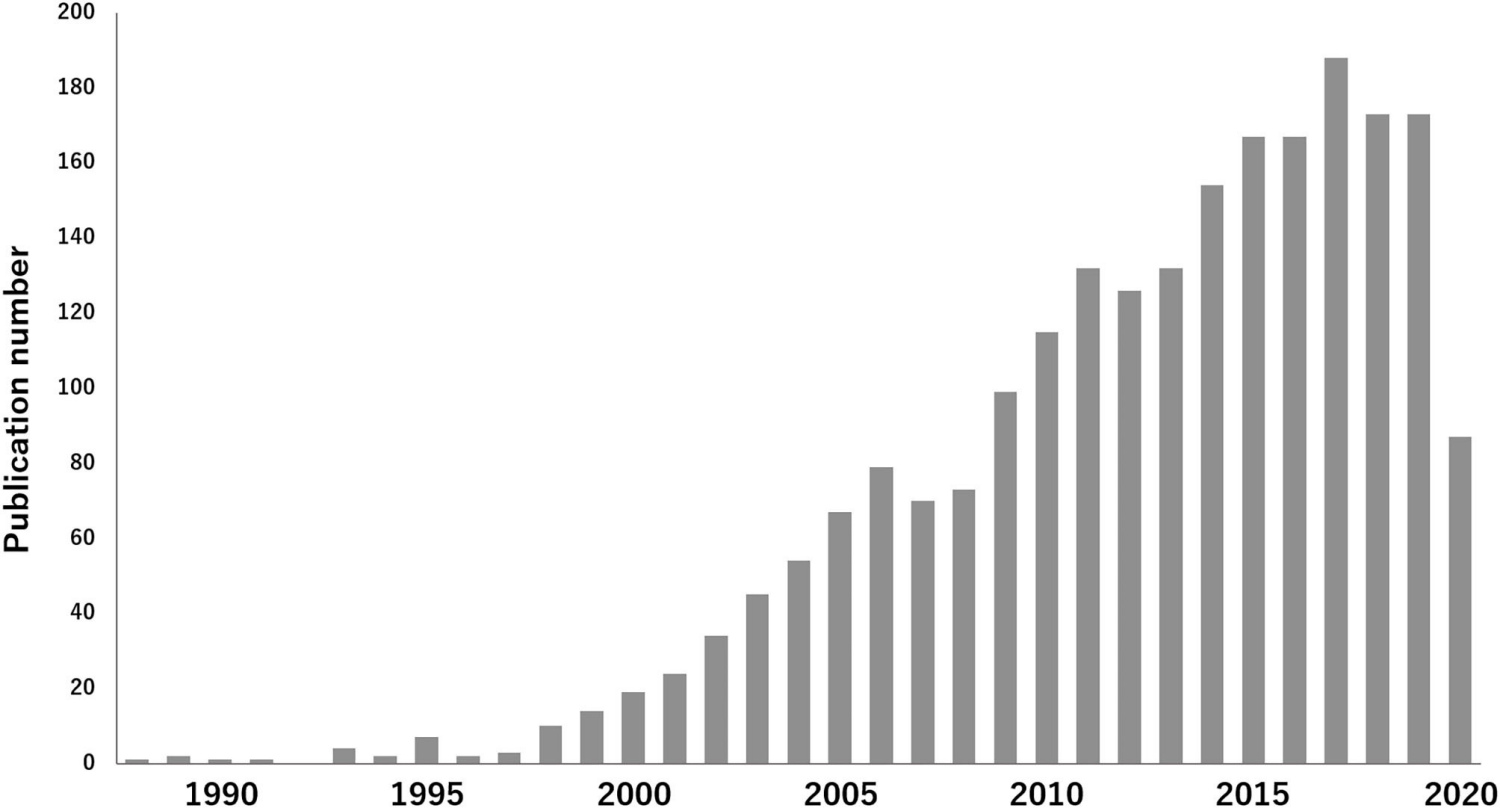
Increase of published articles on TEVG: The interest in research of TEVG research area is visualized. According to a survey conducted at PubMed, 2,226 articles were published until June 2020, using the key words 'tissue engineering and vascular grafts', which demonstrates the increase of published articles in this field.

Within the field of TE, substantial effort has been made to discover ways of overcoming all these limitations of vascular grafts (Figure 1). The concept of tissue-engineered vascular graft (TEVG) is to develop an alternative vascular graft that integrates with the patient's tissue and behaves like a native vascular vessel, including the self-regenerative and growth functions. As TEVG has 3 basic components: (1) a structural scaffold material, (2) cell engrafting and remodeling tissue, and (3) biological cue to recruit and facilitate the assembly of cells, it is a research area which embraces diverse technologies. With the innovation of biomaterials and 3D printing technology, along with stem cell research and an *in vitro* culture system, scaffold-based TEVG has emerged as a promising approach for developing a novel small-caliber graft (1, 24).

In TEVG, the biomaterials are used as scaffolds which mimic the native extracellular matrix (ECM), supporting the growth of cells and guiding tissue regeneration (20, 25). In VTE, the scaffolds should exhibit a variety of characteristics so they can be applied successfully as vessel grafts. The material chosen to produce the scaffold must be biocompatible and not cause an immune response. The vascular scaffolds should exhibit long term patency, an antithrombotic surface and the ability to remodel as a functional tissue (26). Furthermore, the manufacturing technique should provide the formation of a vascular scaffold with pores that guarantee the transport of the metabolic contents and migration of cells (27, 28).

The mechanical properties of vascular scaffolds are also particularly important. These biomaterials should support blood flow and should be able to maintain their structure in the face of physiological stimuli. They must have both elasticity and resistance and be suturable (19, 26). Thus, the evaluation of the mechanical properties of vascular scaffolds is crucial and they should be compatible with the native vessel which is to be repaired. The mechanical properties of scaffolds have been compared to native vessels by various assays, such as the longitudinal tensile test and circumferential tensile testing (29–31). Through these tests, the stress-strain curve can be plotted and parameters such as the ultimate tensile strength, elongation at break and Young's modulus can be recorded (29, 30). The burst pressure and permeability test are mechanical tests that can be used to evaluate the response of the scaffolds in terms of withstanding physiologic pressure (29, 30). The viscoelasticity properties can be analyzed by ball indentation. This test can provide information such as the stiffness, compressibility, and residual force of the scaffolds (32).

Furthermore, vascular scaffolds should establish a tissue organization biomimetic for the native vessels (33). Blood vessels are composed of three layers: the tunica intima, the tunica media, and the tunica adventitia. In the intimate layer, endothelial cells are organized in a monolayer (19, 25). The middle layer is composed predominantly of smooth muscle cells, collagen and elastin fibers, which maintain the mechanical strength, elasticity and vasoactive response (25, 34). The outermost layer, the adventitia, is composed of fibroblasts, which are responsible for preventing the expansion of blood vessels (25). The scaffolds developed by VTE should favor the growth of the cell types found in the vessel wall: endothelial cells, smooth muscle cells and fibroblasts.

## VTE Techniques

**Figure 2:**
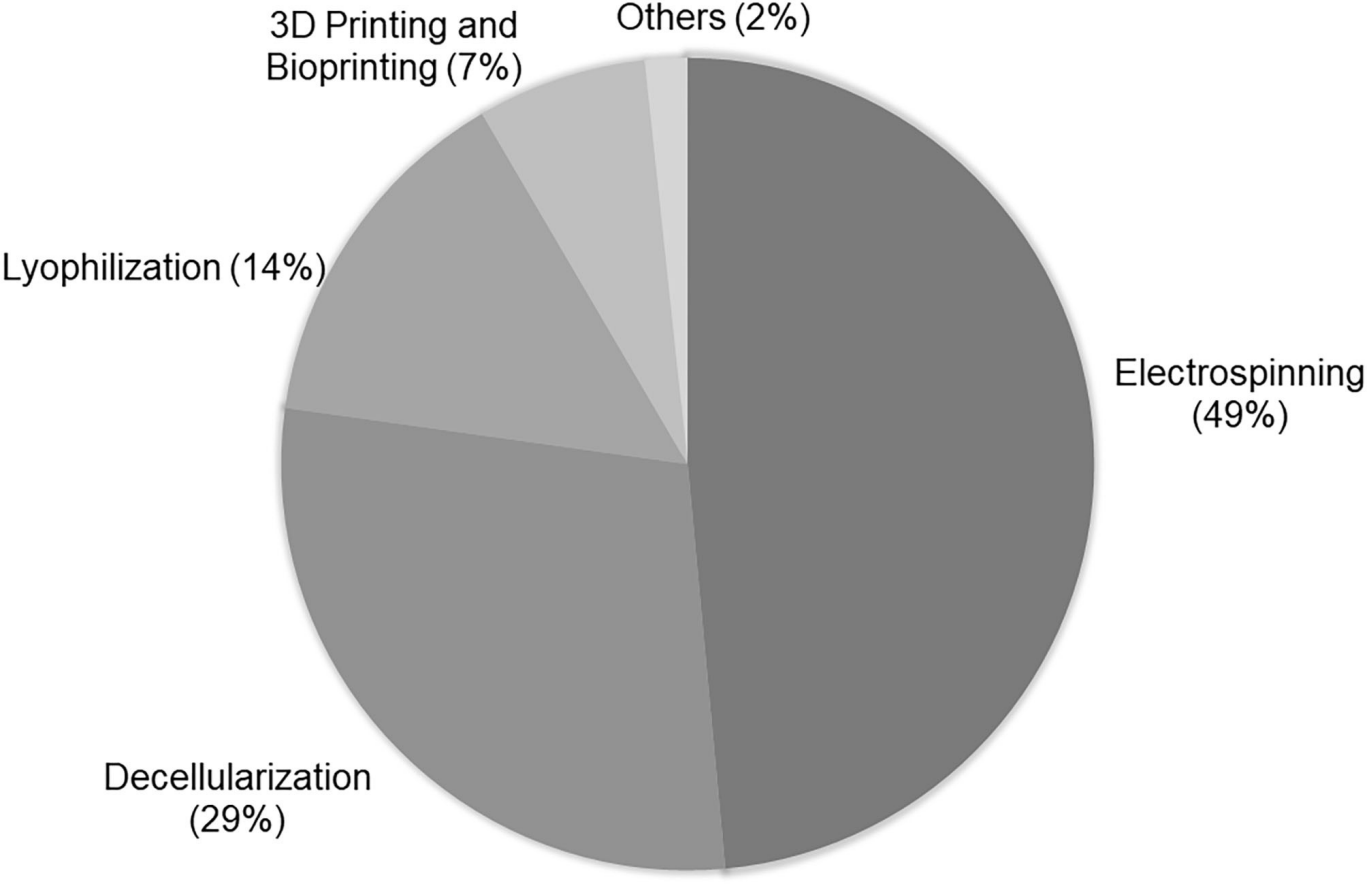
Techniques used in VTE: Percentage of techniques used in vascular tissue engineering according to research realized in PubMed using “tissue engineering and vascular grafts” as terms of search. The research was based on the papers published in the last 5 years. For analysis, the abstracts of original papers were read.

In accordance with a search realized in PubMed for the terms “tissue engineering and vascular grafts,” it is verified that most vascular scaffolds have been obtained by electrospinning, decellularization, lyophilization, and 3D printing and bioprinting (Figure 2).

### Electrospinning

**Figure 3:**
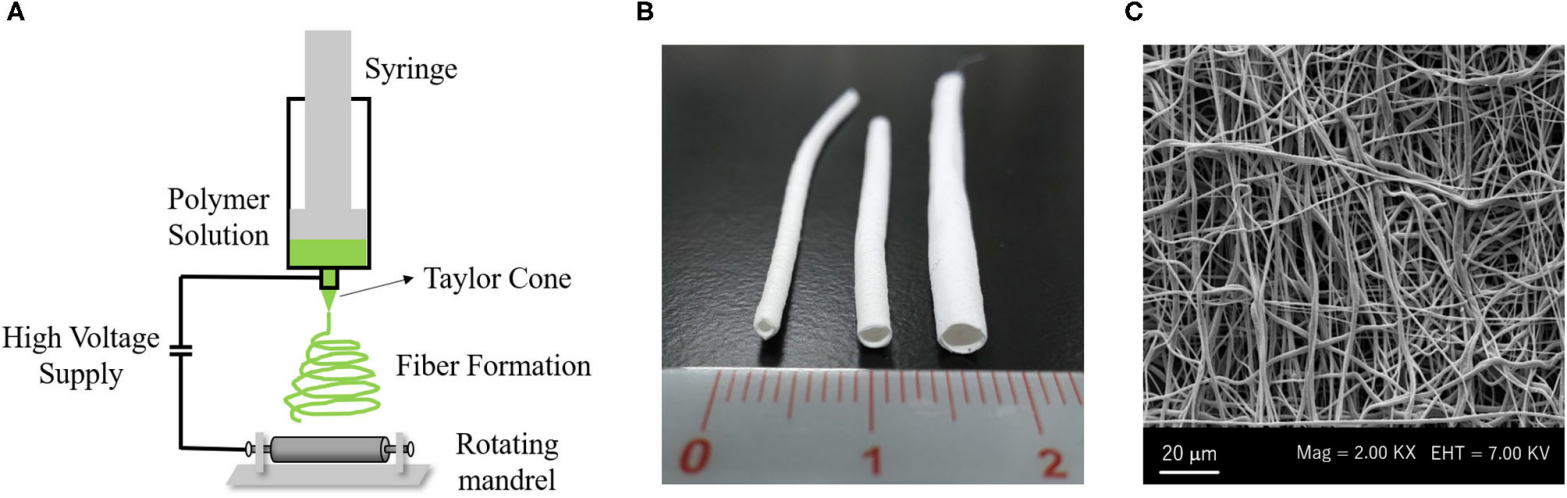
Electrospinning system to create a vascular graft: **(A)** Electrospinning machine consists of a syringe with a needle attached to its tip connected to an electrode, with a high voltage supply and a rotating mandrel as collector plate. **(B)** Macroscopy aspect of PCL vascular graft with small diameter (Left: 1 mm inner diameter, Middle: 2 mm inner diameter, Right: 3 mm inner diameter). **(C)** Scanning electronic microscopy (SEM) image of PCL electrospun fibers. All Images are provided from the authors.

Electrodynamic techniques such as electrospinning are fascinating for developing biomaterials with the necessary characteristics for application in scaffold-based VTE (27). The electrospinning technique allows for the development of three-dimensional scaffolds based on fibers constructed from different polymers, making it possible to control the diameter of the fibers and the porosity of the material (1, 35). The equipment of electrospinning consists of a syringe with a needle attached to its tip connected to an electrode, a hydrostatic pump and an electrical source (36) (Figure 3A). In the syringe, a polymer solution is conditioned and through the hydrostatic pump, it is directed to a collecting plate. When the polymeric solution is released, it is energized by the electrode, which causes a deformation of the drop and, following this, the formation of a conical jet, known as Taylor cone (27, 37). This jet originates the solid fibers that are deposited in the collector plate (38).

The scaffolds developed by the electrospinning technique are formed by fibers that mimic the physical structure of collagen fibers from ECM (Figure 3B), providing a biomimetic microenvironment for cell development and proliferation (1, 35, 39). In addition, the electrospinning technique can develop scaffolds with adequate mechanical properties for vascular application, according to the polymer chosen for their manufacture (1). Many studies have combined synthetic and natural polymers to obtain electrospun scaffolds with mechanical properties similar to those of native vessels and also with good cellular response. Norouzi and colleagues developed a double layer vascular scaffold with polycaprolactone (PCL) associated with heparin. Its Young's modulus was 1.55 MPa, which is similar to that of native arteries. However, the rupture pressure of the scaffold was lower than the autologous grafts, as for example, when the saphenous veins were used as a graft (40).

As previously cited, the porosity and interconnectivity of scaffolds are essential for assistance in cell growth. During their production, therefore, the degree of porosity and the pore size of the scaffolds should be observed. If the scaffolds exhibit small pores and low porosity, they can cause inhibition of cell adhesion, infiltration, and tissue regeneration (41, 42). Electrospinning demonstrates its ability for adapting the pore size, interconnectivity, and fiber size by standardization of the parameters, such as flow rate, tension voltage, and concentration of the polymeric solution (28). Aslani and colleagues developed electrospun scaffolds using poly(lactic acid) (PLA) and acetylsalicylic acid. The scaffolds showed fibers with 1.05–1.30 nm of diameter and a degree of porosity above 43%. Interconnected pores were observed between the fibers, which facilitate the transport of nutrients and metabolic waste (18). Scaffolds with greater porosity have also been developed. Gao and colleagues developed electrospun PCL fibers with 6.51 ± 1.02 μm of diameter and porosity of 81.05 ± 1.38% (20). The authors reported that the high porosity of the scaffolds was satisfactory for their application as vascular grafts. However, excessive porosity can lead to some complications for the vascular grafts, such as excessive bleeding (4) or low mechanical resistance (41).

Scaffolds with a tubular shape can be produced directly by electrospinning, using mandrels as the collecting plate (Figure 3C). Mandrels with varying diameters have been used to develop scaffolds with different diameters which can be applied in a variety of vessels (33, 43). McClure and colleagues used two different diameter mandrels−4 and 80 mm—at a rotation speed of 500–8,000 rpm. In the 80 mm mandrel, it was seen that the high rotation favored the formation of more aligned fibers and produced more rigid scaffolds with less compliance and breaking resistance when compared to the 4 mm mandrel, which showed fibers without alignment. From these tests, the scaffold produced using the 80 mm mandrel would be more interesting for vascular application (44). Fukunishi and colleagues developed PCL/Chitosan electrospun scaffolds using a 1 mm mandrel and implanted them in a mouse model. They obtained very fine and uniform fibers with a diameter of 150 ± 62 nm. The developed vascular graft obtained a lumen diameter equal to the native aorta artery and a successful *in vivo* remodeling after 3 months (45).

The synthetic vascular grafts have some limitations, such as incomplete endothelization, thrombosis, stenosis after implantation and limited function. These limitations can be reduced through the association of growth factors or other bioactive molecules with the scaffolds (46, 47). The scaffolds developed by electrospinning can be associated with biomolecules in different ways, for example, emulsion electrospinning (48), post-electrospinning immobilization, coaxial electrospinning, emulsion electrospinning, and direct blending. The electrospinning technique, as with drug carriers, provides high load capacity, high encapsulation efficiency, ease of operation, and cost-effectiveness, which are attractive features for associating with drugs (49). Therefore, biomolecules such as vascular endothelial growth factor (VEGF), heparin (50, 51), chondroitin sulfate (52), among others, have already been successfully associated with vascular electrospun scaffolds.

The coaxial electrospinning technique is a modification of traditional electrospinning (27), which uses a special spinneret composed of two coaxial capillaries that can simultaneously electrify two different polymer solutions, resulting in a core-shell fiber (39, 53). Various groups have used this method to associate bioactive molecules with scaffolds and to enhance their cellular functionality (47). Coaxial electrospinning can also be used to produce fibers with a natural shell and a mechanically resistant core. Duan and colleagues used coaxial electrospinning to produce PCL/collagen fibers. The core of the fibers was formed from a PCL solution and the shell from collagen. These electrospun scaffolds promoted cell binding and penetration and showed good mechanical properties. They showed a Young's modulus of 2.12 ± 0.05, larger than the native arteries (1.2 MPa) and the veins (0.6 MPa) (39). In another study, Coimbra and colleagues developed vascular scaffolds through coaxial electrospinning using PCL in the core and gelatin in the shell. The presence of gelatin increased the hydrophilicity of the scaffolds, resulting in their heightened performance when in contact with blood. It decreased the hemolytic and thrombogenic character of the materials (53).

### Decellularization

**Figure 4:**
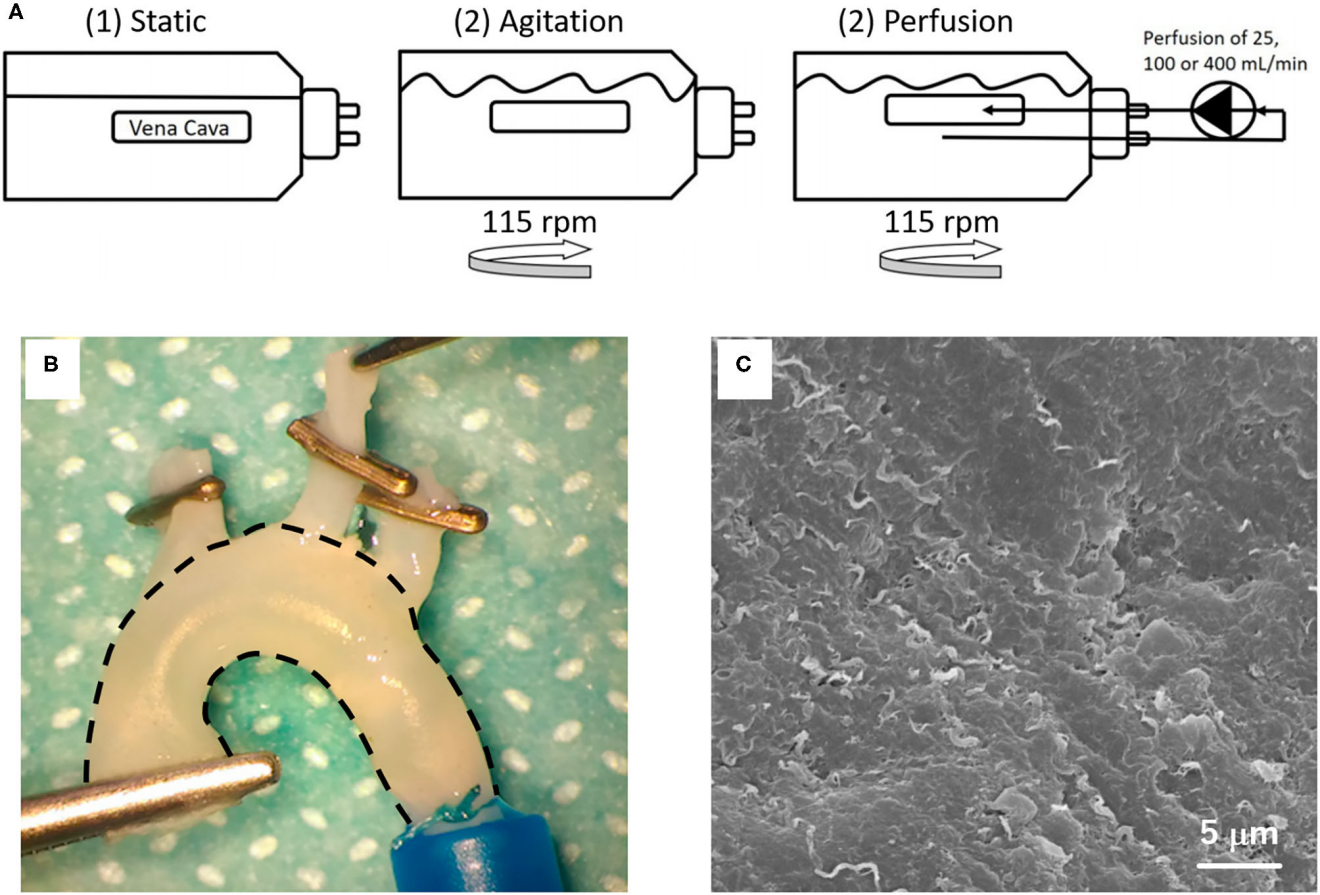
Decellularization methodology: **(A)** Schematic illustration of the decellularization setup of porcine vena cava with detergents (1) under static conditions, (2) by agitation at 115 rpm or (3–5) by perfusion, with agitation at 115 rpm and perfusion at 25, 100, or 400 mL/min. The images are adapted from a report by Simsa et al. (55). **(B)** Macroscopic appearance and **(C)** SEM image of decellularized rat aorta. The image is adapted from a report by Sugimura et al. (56) and Toshmatova et al. (57), respectively.

Decellularization has been the second most used method for the production of vascular scaffolds (Figure 2). Decellularization consists of the total removal of the cells from tissue or an organ and maintenance of the ECM (54) (Figure 4). As the ECM is preserved, the intrinsic characteristics and the tissue shape are maintained (58). Therefore, the scaffolds generated by this methodology are a very promising approach in VTE.

The vessel walls contain mainly collagen, elastin and glycosaminoglycan (GAG), in addition to fibronectin and vitronectin (59). Collagen and elastin provide tensile strength and elasticity for the vascular walls, respectively (34). GAGs are related to viscoelasticity (29, 34). Moreover, GAG also retains growth factors and cytokines (29, 55). Fibronectin and vitronectin are related to interactions between cells and ECM through integrins, which are important for recellularization (55). The preservation of the natural components of vascular ECM, therefore, provides a natural structure and mechanical properties similar to native vessels. In addition, the proteins and GAGs supply different domains for cell adhesion and development (34). Another advantage of decellularized scaffolds is the variety of sources that can be used for their obtention. Decellularization can be conducted from allogenic or xenogenic sources as all cellular and antigen material must be removed, reducing the risk of immune and/or inflammatory response (29).

Various vessels have been decellularized in order to obtain a vascular graft, as for example, the carotid artery (30), aorta artery (32, 60), internal mammary artery (61), umbilical artery (62), saphenous vein (63), coronary artery (64), femoral vein (58), and the vena cava (55). The protocols for vessel decellularization include a number of agents, such as surfactants, enzymes, solvents, and physical methods. Sodium Dodecyl Sulfate (SDS) is an anionic surfactant which is present in most vascular decellularization protocols described in the literature. In addition to SDS, the detergents TritonX-100 (TX) and Tributyl phosphate (TNBP), and the enzymes DNase and trypsin are also widely used. The detergents or surfactants remove the cells from the tissue structure through the solubilization of membranes and dissociation of DNA (29). Meanwhile, the enzymes realize proteolysis. The trypsin acts on native collagen and elastin (65). The nucleases, such as DNase, cleave nucleic acid sequences and can assist in the removal of nucleotides after cell lysis (65). In addition to these agents, hypotonic solutions, such as deionized water, and solvents, such as alcohol and acetone, are also reported in some vascular decellularization protocols.

A great challenge for the decellularization vessel protocols is to combine an ideal concentration of an agent and a sufficient reaction time for the removal of the cells without destruction or alteration of the vascular ECM. In order to maintain the matrix characteristics and ensure a complete removal of cellular content, most of the protocols use a combination of agents to decellularize the vascular tissue. Li and colleagues evaluated a number of protocols to decellularize porcine carotid arteries. They analyzed single-reagent treatments based on SDS, TX, or trypsin. When only SDS (0.1, 0.5, or 1%) was used for 24 or 48 h, they obtained an incomplete nuclei removal. When the time was increased to 72 h, the concentrations of 0.5 and 1% of SDS resulted in complete cell nuclei removal. However, with the increase of SDS concentration, the hematoxylin and eosin (H&E) staining demonstrated more severe damage in the ECM. When Triton-X 1% was used alone (24–72 h), the histoarchitecture of ECM was maintained grossly compact, yet it was not able to remove all the nuclei content. The trypsin (0.1–0.5%) was also unable to completely decellularize the arteries. At the same time, the treatment with trypsin over 6 h caused the disappearance of layer organization of the vessel wall. Based on these results, the authors developed a combined protocol using 0.5% SDS (36 h) followed by 1% Triton-X100 (36 h). Although both are surfactants, SDS acts on non-covalent interactions of the native proteins and Triton-X100 on lipid-lipid and lipid-protein bonds. SDS is, therefore, more effective for decellularization of vascular tissue than Triton-X100. However, as the Triton-X100 does not act on the interactions between proteins, it is less aggressive to ECM, causing full preservation of the structural integrity of arterial tissue. The combination of these two detergents, therefore, resulted in a more effective decellularization of the carotid arteries (29). Although the protocol developed by Li and colleagues was effective in relation to decellularization and less aggressive to the matrix structure, it still caused a reduction in elastin and GAGs content, compared to the native vessel (29). The reduction of the ECM components is expected after decellularization (34). In addition to causing an impact on the biological properties, these changes can also result in losses in the structural integrity and mechanical properties of decellularized vessels (64). Thus, it is important to assess the impact of these changes in *in vitro* mechanical tests and in *in vivo* models.

Some studies also combine physical methods for chemical or biological agents in order to increase the penetration of agents and increase the decellularization of vascular tissue (32, 55, 60, 61). Simsa and colleagues evaluated the decellularization of vena cavas with TritonX-100, TnBP, and DNase under static, agitation, and perfusion conditions. They showed that the three methods were able to reduce the DNA content and similar decellularization efficacy was exhibited. Nevertheless, when the samples were analyzed by scanning electron microscopy, it was observed that the static condition resulted in a greater remnant debris in the luminal surface of the vessel than in the other groups. In this study, the agitation and perfusion in velocity until 100 mL/min was preferable for promoting vessel decellularization (55).

Non-effective decellularization can cause inflammatory response and antigenicity, elevating the risk of failure of a graft (32). It is therefore important to highlight that some criteria must be observed to ensure that the decellularization of tissue can be considered satisfactory. According to Crapo and colleagues, decellularized tissue, including the vessels, should show <50 ng dsDNA/mg ECM dry weight, <200 bp DNA fragment length and lack of visible nuclear material in sections of the tissue stained with DAPI or H&E (65). In addition to demonstrating effectiveness in removing cells and assessing the mechanical properties of tissue after decellularization, the vascular scaffolds should also be evaluated in terms of cytotoxicity. The use of biological and, mainly, chemical agents can generate residual products that can affect further cell growth and development. In vascular tissue, cytotoxicity has been evaluated preferentially with HUVECs (29, 55, 64). Stem cells, such as adipose stem cells (ASC), mesenchymal stromal cells (MSCs) from Wharton's Jelly (62), smooth muscle cells (SMCs) (66) and endothelial progenitor cells have also been reported (64), as well as epithelial cell lines (31). The cytotoxicity has been assessed by metabolic tests, such as Counting Kit-8 assay (CCK-8) (29, 64), Alamar Blue (64), and MTS assay (55).

*In vitro* studies have shown that decellularized vessel scaffolds can support cell adhesion and development. Campbell and colleagues decellularized porcine coronary arteries and conducted their cellularization with bovine aortic ECs and bovine SMCs. They seeded the ECs in the lumen along with the SMCs on the abluminal side and on the lateral edges of the arteries. H&E and DAPI staining showed that the xenogeneic cells could successfully adhere and grow in decellularized tissue and an endothelial layer was formed after 10 days of seeding (66). The promotion of endothelization of scaffolds is crucial for *in vivo* application of the scaffolds and for preventing their failure by thrombi formation. Lin and colleagues evaluated cell adhesion on decellularized porcine coronary arteries. They demonstrated that rat ASCs and HUVECs could adhere and proliferate onto decellularized arteries. The authors also evaluated the *in vivo* endothelization of scaffolds in a rat abdominal aorta repair model. In their study, it was observed that no deaths occurred in the animals which received ASC seeded decellularized arteries. CD31 and von-Willebrand factor (vWF) staining showed the endothelization of the lumen of the implanted seeded scaffolds. However, α-SMC actin staining was also observed, demonstrating intimal hyperplasia (64). In order to increase the success of scaffolds, some research has modified the surface of decellularized vessels. Biomolecules that improve endothelization or that prevent hyperplasia and thrombogenesis have been added to decellularized scaffolds (29, 60, 67). In their study, Dimitrievska and colleagues immobilized the anticoagulant heparin on decellularized aorta arteries and evaluated the adhesion of platelets to their surface. They observed that the number of platelets that adhered to the scaffolds substantially reduced post-heparin immobilization. Therefore, the adhered platelets showed a rounded morphology, that is, they did not exhibit an activated morphology, typically identified by pseudopods. The heparin-decellularized aortas also support the adhesion and proliferation of HUVECs (67).

### Lyophilization

**Figure 5:**
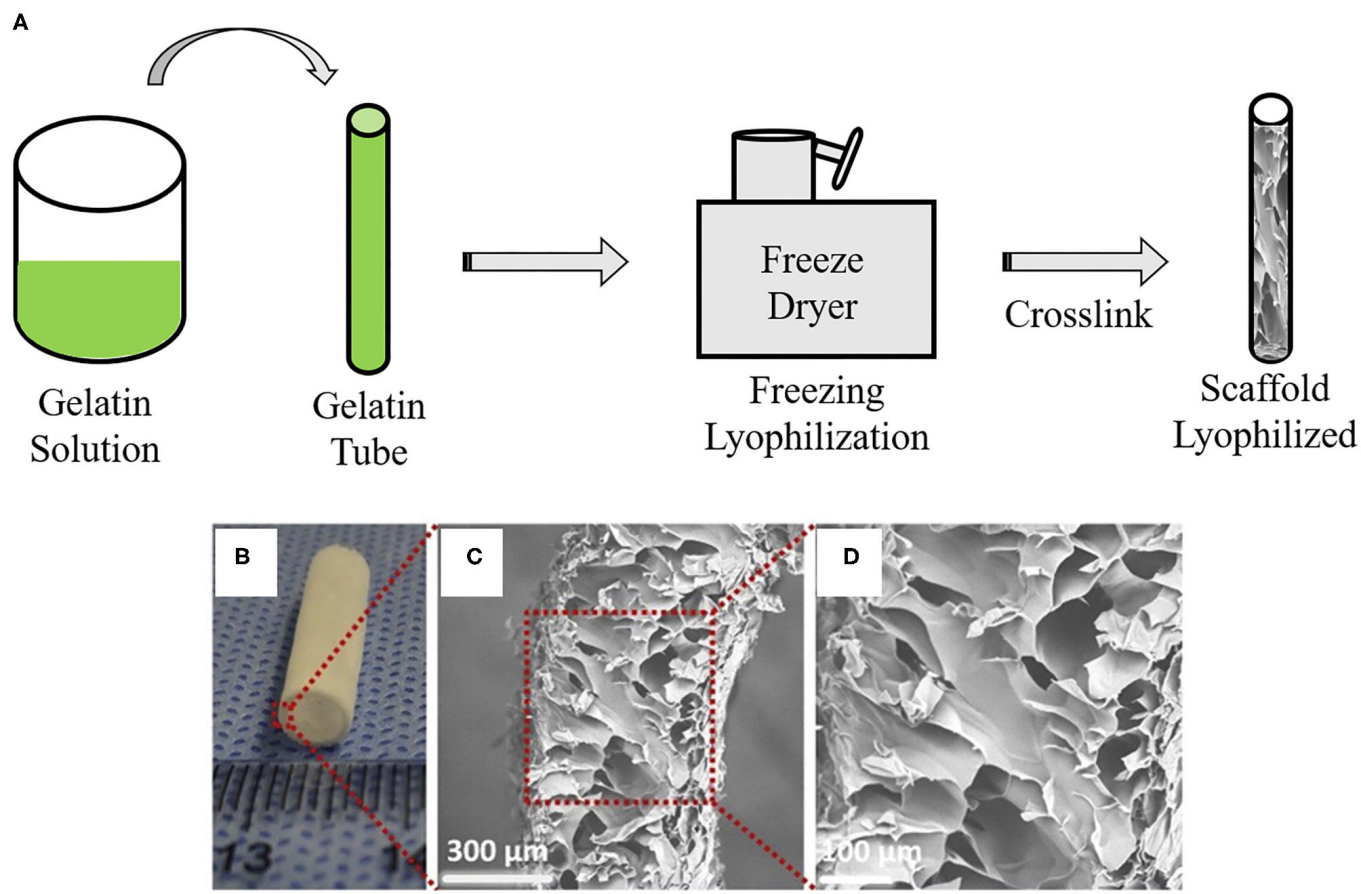
Vascular graft developed by lyophilization: **(A)** Schematic overview of lyophilization method to create gelatin-base vascular graft. Gelatin gel solution material is loaded into a mold cylinder and then freezing and lyophilization are performed using a freeze dryer. Remaining is a lyophilized scaffold. **(B)** Macroscopic appearance and **(C,D)** SEM images of vascular graft made by a lyophilization method. The images are adapted from a report by Yao et al. (68).

Lyophilization is a physical technique characterized by the process of passing an aqueous solution to the solid state through the loss of water from the material at low temperatures (40). In VTE, lyophilization aims to produce a completely dry and stable graft (Figure 5). The lyophilized grafts can easily be sterilized with ethylene oxide or gamma radiation (69). This technique does not change the polymeric properties, such as the mechanical characteristics; it reduces calcification and allows for graft storage for later use (70). Reinhardt and colleagues developed lyophilized vascular scaffolds from poly (glycolic acid) (PGA) and polycaprolactone-co-lactide (PCLA). Firstly, a solution of these polymers was inserted in a cylinder which was snap-frozen at −20°C for 20 min and then lyophilized for 24 h. The authors obtained highly porous scaffolds with an inner diameter of 0.91 mm and wall thickness of 300 μm (71). Wang and colleagues developed poly-L-lactic acid (PLLA), poly(L-lactide-*co*-caprolactone) (PLCL) and poly(lactic-*co*-glycolic acid) (PLGA) scaffolds by dual phase separation technique and lyophilization. The polymer solutions were kept at 60°C under magnetic stirring and phase separated at −80°C for 12 h. Following this, the polymer gels were taken out and immersed in an ice/water mixture to exchange the solvent and then lyophilized for 2 days. These scaffolds showed a macroporous outer layer with 38.4 ± 19.3 μm, which was suitable for SMC infiltration, and an inner layer with a microporous structure, suitable for endothelization. The mechanical tests showed sufficient mechanical properties, such elastic modulus (6.22 ± 1.89 MPa) and compressive modulus (0.98 ± 0.19 MPa), with enough strength to withstand external forces. However, the suture strength of the scaffold (0.27 ± 0.02 N) was less than that of human mammary artery (1.40 ± 0.01 N) and human saphenous vein (1.81 ± 0.02 N) (72). Lyophilization can also be associated with electrospinning in order to obtain a structure with adequate pores size (40). Norouzi and colleagues developed PCL bi-layered scaffolds functionalized with heparin by combining electrospinning and lyophilization. The developed scaffolds showed large pores with an average pore diameter of 289 ± 118 μm. SMCs were seeded and could infiltrate easily in their structure. In addition, the graft had a Young's modulus (1.55 ± 0.32 MPa), which is similar to the coronary artery (1.41 ± 0.72 MPa) (40).

### 3D Printing and Bioprinting

3D printing, also known as additive manufacturing, is an innovative tool in regenerative medicine. In the last few years, this technique has been attracting the interest of several research groups within the TE area due its ability of producing complex materials with considerable reproducibility and low-cost (29, 54). 3D printing comprises the steps of the acquisition of images from the organ or tissue by computed tomography (CT) or magnetic resonance imaging (MRI), the creation of a 3D project using a computer software, selection of a printable ink and fabrication of a 3D solid object layer-by-layer by a 3D printer (73). Despite being applied in different areas of TE, according to the search carried out by this research group, 3D printing has still been little applied for the development of vascular scaffolds (Figure 2). According to a previous study carried out by this research group, 3D printing has been more used for the regeneration of hard tissue such as bone and cartilage (54). The development of small scaffolds with a complex microstructure is still a challenge for this methodology and these factors may contribute to its limited use in VTE. In the vascular area, 3D printing has been used more commonly for the development of 3D anatomical models of vessel networks for surgery training (74, 75).

**Figure 6:**
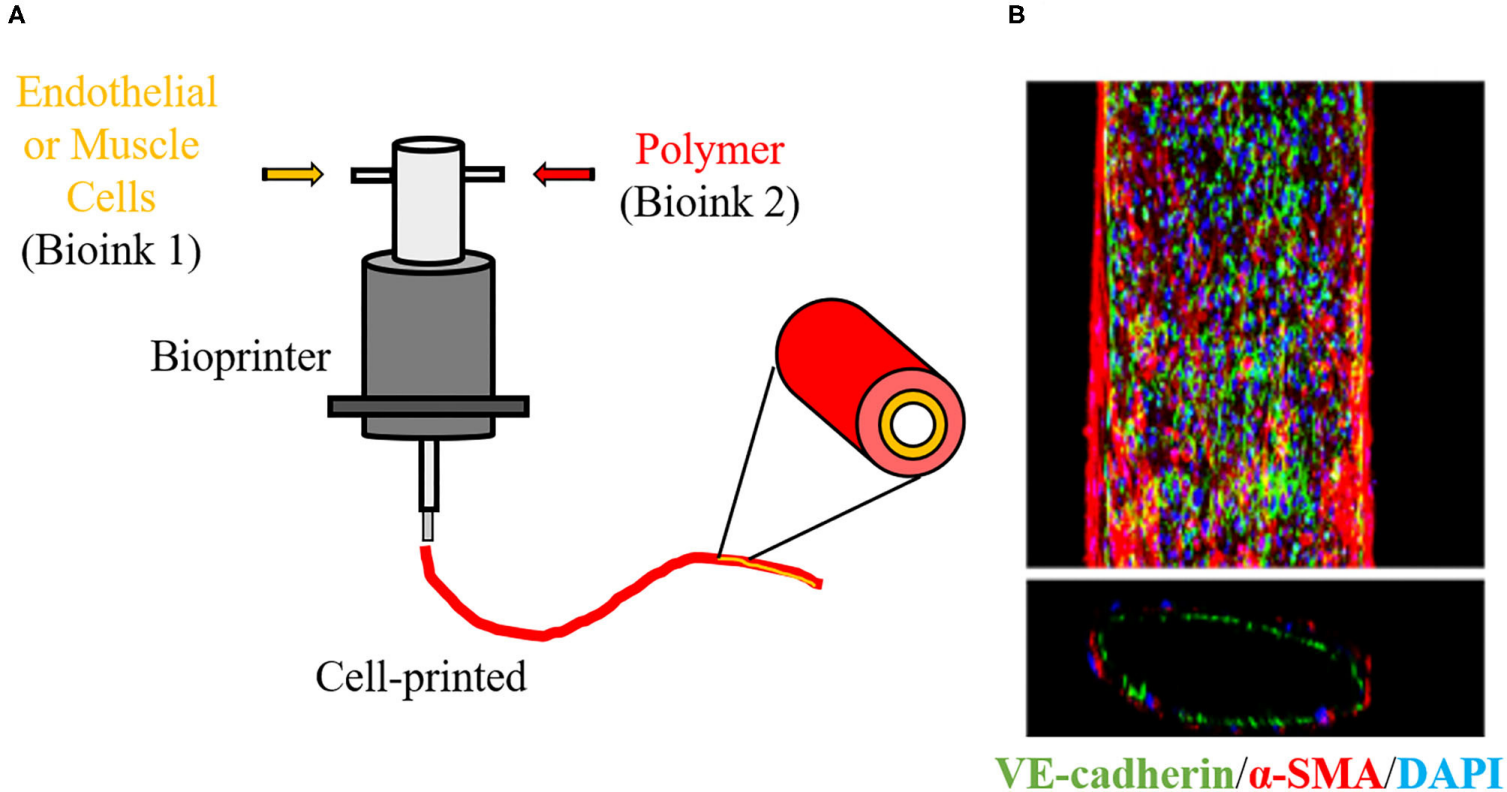
3D Bioprinter. **(A)** Schematic overview of 3D cell printing technique to create a vascular graft using bioinks. 3D bioprinter deposit polymers with living cells layer-by-layer in a desired structure, allowing to create a vascular architecture with endothelial and smooth muscle layers. **(B)** Immunofluorescence staining image show that a 3D-bioprinted vascular graft is constructed of endothelial cells (green) and smooth muscle cells (red). Blue indicates nuclear. The image is adapted from a report by Gao (76).

Despite few groups using 3D printing for the development of vascular scaffolds, its derivative, bioprinting, has already been applied in VTE and represents a large part of the studies reported in Figure 2. Bioprinting is one the most innovative technologies in the TE field. This technology refers to 3D printing with cells in the ink, known as bioink. In bioprinting, the bioink is deposited by a bioprinter, also layer-by-layer, resulting in a scaffold with cells embedded in its structure (Figure 6) (54). This methodology favors the construction of a scaffold with high initial cell loading density and adequate cell distribution (73). As it is mixed with the cells, the polymer used in bioink may be considered biocompatible. In VTE, alginate and gelatin and their derivatives or mixtures of them with other substrates have been widely used for bioprinting (77). Cui and colleagues developed a bi-layered cell vascular construct using a customized 3D bioprinter. To achieve this, they used a coaxial needle extrusion system. A bioink containing gelatin methacrylate (GelMA) and human SMCs was extruded from the external needle and a bioink composed of Pluronic F127, sodium periodate and HUVECs was injected by an internal needle. The newly produced scaffold exhibited a biomimetic vessel structure with smooth muscle and endothelium layers. The authors demonstrated that the viability of SMCs and HUVECs was preserved and that their metabolic activity was increased with *in vitro* culture (78).

A typical restriction of bioprinted scaffolds is their limited mechanical properties. Normally, the polymers used in the bioink do not present great mechanical strength. Freeman and colleagues used gelatin-fibrinogen and human dermal fibroblasts as a bioink to develop a vascular scaffold in a rotatory bioprinter. After bioprinting, this vascular construct was submerged in a bath of thrombin to crosslink the fibrinogen and was then cultivated at 37°C. During cultivation, the gelatin was vacated from the scaffold. The authors demonstrated that the fibroblasts were present in different regions of the construct in the initial culture time. In addition, with the culture, the deposition of collagen in the scaffolds was verified. However, the authors noted that the bioprinted constructs suffered a reduction in their dimensions with the culture, mainly at the beginning of the cultivation period. Moreover, the elastic modulus, burst pressure and ultimate tensile strength were low and only after the *in vitro* cultivation was there an increase in their values. With 60 days of *in vitro* cultivation, the estimated burst pressure of the vascular scaffolds was 1,100 mmHg, which represents about 52% of the human saphenous vein, which is frequently used as a vascular graft (79). The scaffolds normally serve as temporary substrates for the growth of cells and deposition of a matrix. It is therefore expected that with the maturation of these structures, they will show an improvement in their mechanical properties (78, 79). Nevertheless, these aforementioned factors should be considered in the development of other bioprinted vascular grafts.

## Materials Used in VTE

**Figure 7:**
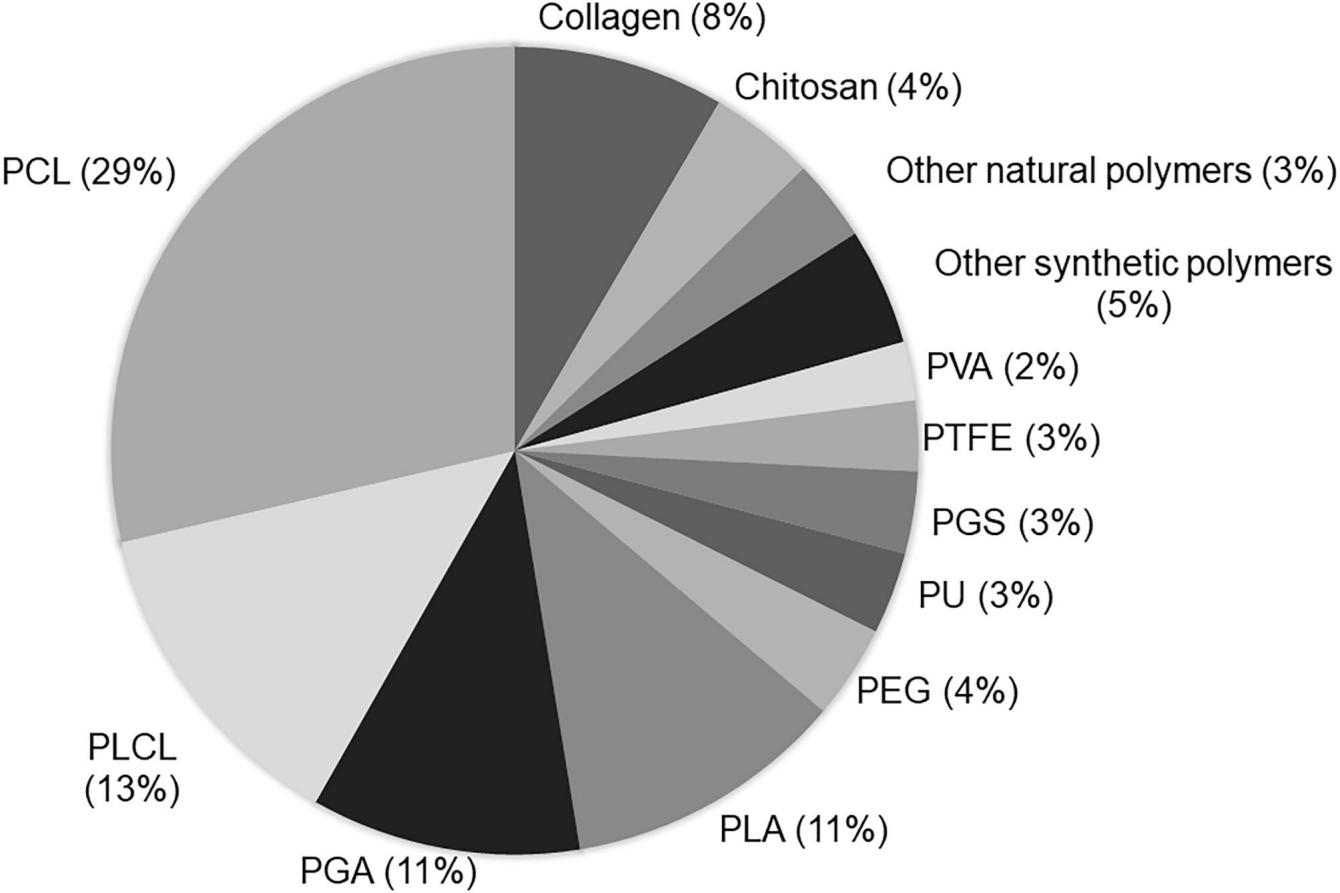
Current biomaterial polymers used to produce vascular scaffolds: The term “tissue engineering and vascular grafts” was used in the PubMed search. The number of papers using each polymer in the last 5 years was calculated. The analysis was based on the reading of the title and/or abstracts of original papers.

Vascular scaffolds have been produced from natural polymers, synthetic polymers or decellularized matrices (51). Among the polymers, the synthetic polyesters such as PCL, PLA, PGA, and PLCL, have been used mainly in VTE (Figure 7). A great advantage of synthetic polymers is the fact that their characteristics can be adjusted according to clinical needs, such as the mechanical properties and degradation rate (43). However, these polymers have some limitations, such as the lack of natural domains, which are important for cell adhesion and proliferation (1).

Among natural polymers, collagen has been used more widely in VTE (Figure 4). In addition to collagen, elastin, fibrin and chitosan have also been researched for the development of vascular scaffolds. Natural polymers present properties similar to those of the native ECM in terms of functional groups (43). They exhibit natural domains that support cell recognition and adhesion. Because of this, their biological characteristics are usually superior to those of synthetic polymers. However, natural polymers exhibit a greater instability and difficult handling for the production of scaffolds. In addition, natural polymers have poor mechanical properties and rapid degradation (1, 80). In order to improve the scaffold properties, combinations of polymers have been frequently used in VTE.

### Synthetic Polymers

#### PCL

PCL is the most commonly used polymer in VTE. In addition to its biocompatibility and biodegradability, this polyester exhibits slow degradation, low cost and high stability during processing and storage (35, 39). Moreover, the mechanical properties of PCL are determinants for its wide use in the development of vascular scaffolds (35). Gao and colleagues reported that the mechanical properties of PCL, developed by the electrospinning technique, exceed those of natural vessels. For example, the results showed that the maximum stress of PCL scaffolds was 3 MPa while the femoral artery was 1–2 MPa (20). Tan and colleagues developed two groups of electrospun scaffolds: a PCL monolayer and a PCL and PEG bilayer. The monolayer and biolayer scaffold showed Young's modulus of 4.07 and 4.55 MPa and a tensile strength of 1.39 ± 0.29 MPa and 1.93 ± 0.36 MPa, respectively. The study demonstrated that the scaffolds showed appropriate mechanical properties for *in vivo* implantation. In addition, the presence of PEG increased the porosity of the scaffolds, which favors cell proliferation on the inner-layer surface of the scaffold (81). Despite its adequate mechanical properties for VTE application, PCL has hydrophobic characteristics, which can interfere in cell adhesion and proliferation (19, 50). This characteristic can also cause platelet aggregation and intimal hyperplasia of the scaffolds, leading to the failure of vascular grafts (82, 83). In order to improve the biological properties of scaffolds produced from PCL, some authors have combined PCL with other polymers, such as natural polymers, or have functionalized the scaffold surface with biomolecules (82). Radakovic and colleagues used PCL associated with collagen to develop vascular scaffolds. This group demonstrated that the presence of collagen in the scaffolds reduced some mechanical properties, such as maximum elongation and Young's modulus. However, when human microvascular endothelial cells were seeded on their surface, the PCL/collagen scaffolds represented a suitable substrate for cell attachment and growth of these cells (84). In another study, PCL electrospun scaffolds were functionalized with heparin and vascular endothelial growth factor (VEGF). The mechanical properties of the scaffolds were not changed with the functionalization and they were compatible for vascular use. The presence of heparin prevents coagulation of the blood and the VEGF favors the development of endothelial progenitor cells on the scaffold surface (19).

#### PLCL

PLCL is a copolymer of lactic acid and caprolactone. In the copolymer, the brittle behavior of PLA and the low level of stiffness of PCL are adjusted (85). PLCL exhibits slow biodegradation, biocompatibility and elasticity (86). Horakova and colleagues developed a PLCL vascular graft through electrospinning. The scaffold promoted *in vitro* adhesion and proliferation of the endothelial cells. When they were implanted in the *in vivo* model, the scaffolds demonstrated endothelization and presence of collagen fibers in their lumen and favored vessel regeneration. However, 6 months after implantation, it was observed that the scaffolds showed a rapid rate of degradation and loss of mechanical properties. The authors reported that the grafts should have a greater thickness of the vessel wall to maintain the mechanical properties and the correct functioning of the biomaterial (87). Shafiq and colleagues developed a vascular graft through the electrospinning technique using PLCL functionalized with heparin and substance P, a neuropeptide involved in neuroinflammation, cell proliferation and wound healing. These scaffolds were evaluated in an i*n vivo* model. The results of histological analysis demonstrated the formation of new tissue, deposition of collagen and elastin, and a large number of blood vessels. In addition, the graft had slow degradation and adequate mechano-elasticity properties, suggesting that these scaffolds could improve vascular regeneration (88).

#### PGA

PGA is a biodegradable polyester which presents a high level of crystallinity (27), flexibility and lack of inflammatory response *in vivo* (89). However, the degradation rate of PGA is around 6–8 weeks, too fast for vascular clinical applications. For this reason, PGA is generally used in association with other polymers (90). Melchiorri and colleagues developed a vascular graft of PGA/PLA and poly (DL-caprolactone-co-lactic acid) (PCLLA), through electrospinning. The scaffolds showed a tensile strength higher than the native vessel: 2.93 ± 0.26 MPa against 2.2 ± 0.2 MPa of human saphenous vein (91, 92). Fukunishi and colleagues developed scaffolds with PGA and PLCL by combining 3D printing and electrospinning. In the mechanical tests, the burst pressure was no different from the native vessel and compliance was significantly greater. When the scaffolds were implanted *in vivo*, they showed a satisfactory remodeling after 6 months post-operation (91).

#### PLA

PLA exhibits slow degradation and decomposes into lactic acid, processable by the TCA cycle. It is non-immunogenic and is an FDA approved polyester. In VTE, the scaffolds produced only with PLA have not shown positive results. PLA exhibits a hydrophobic structure, making cell development difficult (93). For this reason, PLLA has been associated with other polymers or functionalized for VTE applications (93). Caracciolo and colleagues developed PLLA electrospun scaffolds and functionalized them with heparin. The contact angle measurements demonstrated increased hydrophilicity after functionalization of the scaffolds with heparin, which favored adhesion and proliferation of the human adipose-derived stem cells. Heparin also increases the attachment and infiltration of endothelial cells (94). Fiqriant and colleagues developed PLLA scaffolds functionalized with chitosan and collagen through electrospinning. The authors demonstrated that the tensile strength and burst pressure of the scaffolds were similar to that of native vessels. The biological tests indicated that the developed vascular scaffold exhibits high hemocompatibility, low cytotoxicity, and promotes good cell viability.

#### PTFE

PTFE was one of the polymers used in the development of the first synthetic vascular graft. It is widely used in VTE for the development of grafts of a medium or large diameter, with a few of them being used for <6 mm scaffolds. The small diameter PTFE scaffolds show a high failure rate due to the formation of thrombi within them (95). These events occur because of the high hydrophobicity of PTFE, which favors platelet adhesion and activation of the coagulation cascade. Authors also report that PTFE presents a low cell adhesion rate and proliferation (96). Alternatively, Chen and colleagues developed PTFE scaffolds with ECM and CD34 monoclonal antibodies. The presence of antibodies improved the hydrophilicity and decreased platelet adhesion on the scaffolds. In addition, the association of antibodies and ECM facilitated endothelization, adhesion and proliferation of endothelial cells (95). In another study, PTFE was associated with PEG. The PEG increased the hydrophilicity of the biomaterials and the association of the polymers provoked an increase in cell proliferation (96).

### Natural Polymers

#### Collagen

As previously cited, collagen is the most used natural polymer in VTE. Collagen is the main component of the native ECM (25). Characteristics such as low antigenicity, high biocompatibility, biodegradability, and support for cell adhesion have highly encouraged the use of collagen in the production of vascular scaffolds (4, 39). However, as previously cited, collagen, as with other natural polymers, exhibits poor mechanical properties, and a fast degradation rate, which are limitations for VTE application. Zhang and colleagues used collagen to develop a vascular graft combining electrochemically aligned collagen filaments and electrospinning by circular knitting. The collagen increased the graft rupture force, considered greater than that of the human artery and saphenous vein. The dynamic compliance obtained was similar to that of the human saphenous vein and lower than the human artery when using a PLA scaffold for comparison. In biological tests, collagen provides an increase of adhesion and proliferation of HUVECs when compared to PLA scaffolds (97). Bertram and colleagues used collagen associated with PCL to develop vascular biomaterials through electrospinning. The percentage of associated collagen ranged from 5 to 75%. The authors demonstrated that collagen promotes an increase of endothelial cell viability in all the rated groups after 24 h cultivation. The proportion of 25% collagen showed a reasonably constant cell proliferation rate throughout all the tests, which is considered to be ideal for VTE (35).

#### Chitosan

Chitosan is the second most used natural polymer in VTE. It is derived from chitin through its alkaline deacetylation. Chitosan scaffolds inhibit inflammation during implantation, modify viability chemically and have high affinity with *in vivo* macromolecules (98). In addition, it has been reported that chitosan exhibits anticoagulant properties, low toxicity, and good degradability, which are also interesting for VTE application. Some authors have shown that chitosan exhibits a structure similar to components of ECM, such as glycosaminoglycans (99, 100). This characteristic is important in vascular regeneration due to its inhibitory effects on vascular smooth muscle cells and anticoagulant properties (99, 100). Aussel and colleagues developed a vascular graft with a new formulation of chitosan to assess the potential of mechanical properties for a vascular graft. The new formulation consisted of adopting two different gelation processes: aqueous NaOH induced-gelation and gaseous NH_3_-induced gelation. Moreover, the product was washed several times with deionized water to recover neutral pH and the obtained gels were concentrated at 5 and 10% chitosan. The gels with high concentration (10%) of chitosan showed an increase in elastic module and average resistance, being considered a vascular graft with mechanical properties suitable for *in vivo* implants (101).

#### Elastin

Elastin is a natural elastic polymer. This hydrophilic protein surrounds the internal lamina of arteries and maintains the elasticity of their walls under blood pressure (102). Nguyen and colleagues developed a vascular graft from elastin associated with collagen. The presence of elastin provided an organization and alignment of the collagen fibers similar to ECM. In addition, when SMCs were cultivated onto the scaffolds, the elastin was able to promote their contractility. The authors also reported that the mechanical properties of developed scaffolds improved after cell cultivation, being similar to native vessels (103).

## TEVG *in vivo*

The common concept of TEVG is the development of an autologous tissue-like vascular graft which integrates with the patient's tissue and functions in the same manner as native vasculatures. Although there is a variety of approaches including the use of stem cells (ESC, iPS cells, MSC, autologous cells, etc.), biotube technology and decellularized tissue, in this section we focus on biodegradable synthetic/natural polymers and summarize recent studies of small-caliber *in vivo* TEVG.

## Polymer Base Tevg in Animal Studies

### Synthetic Polymers

#### PCL

PCL is a biodegradable material which has been widely investigated in a variety of tissue engineering research. Pektok and colleagues compared the performance of PCL grafts with ePTFE grafts in rat systemic arterial circulation. Despite the same patency rates between the PCL grafts and the ePTFE grafts during the 24 weeks follow-up period, the PCL grafts demonstrated significantly faster endothelization, extracellular matrix formation and neoangiogenesis, as well as a less consequential stenosis-free rate (104). The excellent biocompatibility and valid revascularization have been proven for PCL grafts by independent research groups; however, Valence and colleagues reported an interesting finding about long term performance of PCL grafts. They implanted electrospun PCL grafts into the rat abdominal aorta, and the observed performance was excellent in terms of patency rate, integrity, and revascularization with limited intimal hyperplasia in the first 6 months. However, the regression of engrafted cells and capillaries as well as the appearance of chondroid metaplasia was observed in the following 12–18 months. Involvement of aging was discussed in the article as the period of 12–18 months represents the entire lifespan of rats, but it is not clear if the aging process could be a contributory factor for tissue regression in the PCL graft (105). Because clinical use of synthetic vascular grafts would be applied mainly to aged patients, it is essential to test using animal models which have a longer life span and to understand the mechanisms underlying the regenerated tissue regression.

#### PLLA

PLLA is another polymer extensively investigated in this field. Because PLLA degrades into lactic acid in living bodies by a non-enzymatic hydrolytic process and is harmlessly excreted (106), it has been clinically applied for a drug delivery system. While PLLA has desirable mechanical properties, its hydrophobic character puts PLLA at a disadvantage as a vascular graft due to early thrombogenicity. PLLA has, therefore, been researched to combine with other materials, including cells or bioactive molecules, taking advantage of its structural properties and biocompatibility. The graft of poly PLCL, a blended polymer of PCL and PLLA, demonstrated patency over an 8 weeks period and favorable revascularization without any structural failure in rodent abdominal aortic interposition conduit models (107). A surface coating with stromal cell-derived factor-1α/heparin or seeding of bone marrow mesenchymal stem cells improved the patency up to 12 weeks and accelerated tissue regeneration without intimal thickening (108, 109).

#### PGA

PGA degrades in a shorter period than PCL or PLA. While this characteristic provides an advantage in tissue remodeling, PGA alone degrades too first and loses its mechanical strength before the remodeled tissue obtains sufficient durability, resulting in graft fracture or aneurysmal formation. Roh and colleagues reported that the PGA-P(CL/LA) scaffold functioned as venous and arterial interposition grafts in mouse models over several weeks despite a rapid decrease in tensile strength, indicating that tissue remodeling was sufficient and, consequently, mechanical property was maintained (110). Other groups demonstrated complete 1-year patency of a PGA/PLLA graft with regeneration of endothelial cells, smooth muscle cells, elastin and collagen fibers in a canine carotid artery (111) and porcine aortic interposition models (112).

#### Poly (Glycerol-Co-Sebacate) (PGS)

PGS is an elastic, flexible but rapidly biodegradable elastomer (113). Khosravi and colleagues prepared the grafts of PGS core and PCL outer sheath and implanted them as infrarenal aortic interposition grafts in mice. The grafts demonstrated excellent 1-year patency without any stenosis or aneurysmal formation. Although an inflammatory response against the residual polymer persisted up to 1-year endpoint, functional vascular regeneration, including organized endothelial cells and contractile smooth muscle cells, was observed (114).

### Natural Polymers

#### Collagen

Collagen is the main component of ECM in animals and has the function of enhancing cell adhesion and proliferation. With these features, substantial research on collagen-containing vascular grafts has been conducted, including clinically applied grafts; collagen-coated PET grafts prevented porous-related bleeding (115) and multilayered collagen scaffold modified to a constant release of basic fibroblast growth factor (bFGF) provided an increase in neoangiogenesis (116). Tillman and colleagues implanted the PCL-collagen compound graft (weight ration = 1:1) as an aorta-iliac artery bypass in a rabbit and demonstrated 87.5% of patency rates and sustained mechanical strength in 1 month (117). Grus and colleagues reported that collagen-based three-layer graft (collagen-polyester-collagen) maintained patency even in low-flow conditions in sheep models (118). These findings suggest that collagen is a promising material when combined with other materials or bioactive molecules, even though collagen itself has inadequate mechanical strength and thrombogenicity.

#### Elastin

Elastin is also the predominant component of ECM and provides recoiling energy to the arterial wall. Wise and colleagues reported that two-layer grafts composed of recombinant human tropoelastin and PCL demonstrated good patency and mechanical properties in 1 month, while durability of the graft was similar to that of the human internal mammary artery tested in a rabbit carotid artery interposition model (119). Despite the significant role of elastin in aortic morphogenesis, including the prevention of smooth muscle cell proliferation and intimal hyperplasia in native tissue (120), a limited amount of *in vivo* research on elastin-containing vascular scaffolds has been reported to date.

#### Chitosan

Chitosan is a natural polysaccharide derived from shells and has high biocompatibility as well as favorable biodegradability and antibacterial properties. Using chitosan, Yin and colleagues made a bi-layered graft with PEGylated-chitosan and PLCL and implanted the graft into a canine femoral artery. Twenty-four weeks after implantation, the scaffold was degraded and autologous artery-like tissue regeneration was observed, representing the arranged intima, smooth muscle layers, and connective tissue as neovessels (121). Despite the reported outcomes showing the excellent structural integrity and hemocompatibility as well as identical vascular remodeling with little calcification, follow-up studies will be required as chitosan is a relatively new biomaterial and available information is limited *in vivo*.

#### Silk

Silk fibroin is mechanically fabricated due to its excellent biocompatibility and non-toxic properties. These features are attractive for the utilization in the field of tissue engineering. Cattaneo and colleagues reported that elastic lamina and vasa vasorum, as well as endothelial cells and smooth muscle cells, were formed in the electrospun silk-fibroin grafts within just 7 days after the implantation in a rat abdominal aorta (122). Furthermore, the silk-based graft demonstrated high patency rates (95%/6 months and 85.1%/1-year, respectively) which were significantly higher than those of the ePTFE graft (30%/1-year) (123, 124). A rapid suppression of acute inflammatory response was also reported in these articles.

## TEVG in Human Clinical Trial

### Arterial Model

The first clinical trial of TEVG in arterial circulation was conducted by Lawson and her colleagues (125). Human smooth muscle cells were harvested from uncomplicated donors and expanded *in vitro*. The expanded cells were then seeded onto PGA scaffolds, cultivated in a bioreactor with pulsatile circuit distension for 8 weeks and the developed grafts were then decellularized. Resulting human acellular TEVGs (6 mm in diameter) were implanted in the arms of patients with end-stage renal disease, as an access of hemodialysis. The grafts were implanted in 60 patients from 2012 to 2014. Over minimum 1-year follow up periods, the primary patency rates were 63, 28, 18, and 15% at 6, 12, 18, and 24 months, respectively, which was less satisfactory, even though little evidence of degenerative change, immune reactions, or infections were observed. This trial demonstrated that human acellular TEVG had sufficient mechanical properties and minimal antigenicity in human arterial circulation; however, modifications and improvement will be necessary to apply the TEVG into clinically practical use where patients have multiple underlying diseases and severe systemic arteriosclerosis.

### Venous Model

The most desirable characteristics of TEVGs is that regenerated tissue can integrate and grow with the surrounding host tissues. Hibino and colleagues started the first-in-human trial of TEVGs in repair of congenital heart disease in 2001 (126). They seeded autologous bone marrow-derived mononuclear cells onto biodegradable scaffolds (12–24 mm in diameter) composed of PLCL and PGA/PLA and implanted them as extracardiac cavopulmonary conduits in patients with single ventricle physiology. During the follow up periods (5.8 years, mean), no significant graft dysfunctions, including aneurysmal formation or rupture was observed. Excluding the death from inevitable cardiovascular events and a few interventions for stenosis sites, the TEVGs functioned in large-caliber venous circulation without major failures.

TEVG have only been developed for a few decades and the efficacy of TEVG in human systemic circulation is still unknown, However, Shinoka and colleagues have clearly demonstrated proof of the positive concept of TEVG.

## Summary

The first successful TEVG in a human clinical trial was an autologous cell-seeded degradable scaffold, which represents an encouraging step in this field. Recent advancement of cell culture systems, such as bioreactors and 3D bioprinting technology, has inspired cell based TEVG and showed successful outcomes. However, cell based TEVG carries the risk of rejection and is also time and cost consuming even if patient derived MSC is used to avoid rejection, which limits clinical versatility of the treatment. Because of the urgency of cardiac surgery and the huge number of patients with different backgrounds, the most demanded and desired TEVG, from a cardiac surgeon's point of view, would be a versatile off-the-shelf, small caliber graft. Although this goal is challenging, cell-free scaffold-based application is a promising approach as a variety of biocompatible polymers are now available. In addition to synthetic polymers, and his group have recently developed a method to create a cell-free textile using *in vitro* normal human fibroblast culture system (127). The human derived biomaterial is high compatible with human tissue and has introduced a novel approach in this field of VTE. In addition to exploring new materials, it would be important to understand the process and mechanisms of how currently developed vascular grafts fail and degenerate. Assuming that small caliber vascular grafts are used in patients with diabetic disease and/or atherosclerosis, it is especially important that studies be made in a disease model testing functionality of TEVG as these are generally missing in this area. Because the final goal is to develop a clinical application, a mechanistic study, which is not currently in progress, could open a new avenue of TEVG innovation.

There is no single material which has been approved for clinical use yet, each of them has their own unique features and properties, as reviewed in this paper. Taking advantage of this diversity, a combination of these polymers as well as scaffold modifications with biomolecules and functional cells, would be essential, and further research and optimization will allow us to create ideal and practical small caliber vascular grafts.

## Author Contributions

BL participated in the preparation of the figures and was responsible for writing the abstract, introduction, vascular tissue engineering (VTE), techniques, and polymers used in the VTE. DB participated in the preparation of the figures, responsible for writing the techniques used in the VTE and assisted in the final review of the polymer topics. NW and HK participated in the preparation of figures and writing. KO participated in the preparation of figures, writing, and design of the article. NW, HK, and KO were responsible for writing the introduction (vascular grafts), and *in vivo* studies, including polymers used in animal and human studies. PP conceived the review article, participated in its design and coordination, and carried out the final review of the entire manuscript. All authors read and approved the final manuscript.

## Conflict of Interest

The authors declare that the research was conducted in the absence of any commercial or financial relationships that could be construed as a potential conflict of interest.
